# Cross-cultural comparison of correlates of quality of life and health status: the Whitehall II Study (UK) and the Western New York Health Study (US)

**DOI:** 10.1007/s10654-012-9664-z

**Published:** 2012-03-04

**Authors:** Oscar H. Franco, Yim Lun Wong, Ngianga-Bakwin Kandala, Jane E. Ferrie, Joan M. Dorn, Mika Kivimäki, Aileen Clarke, Richard P. Donahue, Archana Singh Manoux, Jo L. Freudenheim, Maurizio Trevisan, Saverio Stranges

**Affiliations:** 1 Department of Public Health and Primary Care, University of Cambridge, Cambridge, UK; 2 Department of Epidemiology, Erasmus Medical Center, Office Ee21-79, P.O. Box 2040, 3000 CA Rotterdam, The Netherlands; 3 Health Sciences Research Institute, University of Warwick Medical School, Coventry, UK; 4 Department of Epidemiology and Public Health, University College London, London, UK; 5 Department of Exercise and Nutrition Sciences, State University of New York at Buffalo, Buffalo, NY USA; 6 Department of Social and Preventive Medicine, State University of New York at Buffalo, Buffalo, NY USA; 7 Centre for Research in Epidemiology and Population Health, INSERM, Paris, France; 8 Health Sciences System of the Nevada System of Higher Education, Las Vegas, NV USA

**Keywords:** Quality of life, Health status, Sleep, Depressive symptoms, Cross-cultural comparison, Epidemiology, SF-36

## Abstract

Measures of quality of life (QoL) have been found to be predictors of mortality and morbidity; however, there is still limited understanding of the multifaceted nature of these measures and of potential correlates. Using two large populations from the UK and US, we aimed to evaluate and compare measured levels of QoL and the key factors correlated with these levels. Participants were 6,472 white subjects (1,829 women) from the Whitehall II Study (mean age 55.8 years) and 3,684 white subjects (1,903 women) from the Western New York Health Study (mean age 58.7 years). QoL was assessed in both using the physical and mental health component summaries of the short form-36 questionnaire (SF-36). Analysis of covariance was used to compare gender-specific mean scores for the two populations across several potential correlates (including socio-demographic, lifestyle and co-morbidity factors). Levels of reported physical QoL tended to be higher in the UK population (51.2 vs. 48.6) while mental QoL was higher in the US group (53.1 vs. 51.1). Age, sleep duration and depressive symptoms were the main factors correlated with both physical and mental QoL in both samples. Increasing age was associated with poorer physical health but higher mental health scores in both populations (*P* < 0.001). Sleep duration below 6 or above 8 h was associated with lower levels of QoL. Depressive symptoms were strongly associated with poorer mental health scores (*P* < 0.001) while higher BMI, lower physical activity levels and presence of cardiovascular disease were associated with poorer physical health in both samples and gender (*P* < 0.05). There were consistent findings for correlates of QoL in this cross-cultural comparison of two populations from the UK and US. Strongest associations were between lifestyle and co-morbidity factors and the physical health component of the SF-36 rather than the mental health component. This is a novel finding which warrants further consideration.

## Introduction

Self assessed measures of quality of life (QoL) and health status have been associated with development of disease, disability and mortality, and are now considered as key parameters in the process of policy making, allocation of services and provision of care [1–3]. These measures capture a multidimensional perspective of *an individual*’*s* state of health and wellbeing and therefore incorporate a comprehensive definition of health as defined by the World Health Organisation: ‘a complete state of physical, mental and social well-being, and not merely the absence of disease or infirmity’ [4].

Different studies have suggested that self assessed QoL and health status are modified by different factors including age, education, physical activity and depressive symptoms [1, 5–8]. Nevertheless, the majority of available measures of QoL yield results that are generally culture-specific and lack validation in multiple populations, except for a few, which include the short form 36 (SF-36) [9]. These challenges in optimally measuring QoL have limited the capacity to understand the interplay *between personal and social* factors with QoL and how this might vary across populations.

Hence, in this report, we performed a cross-cultural comparison of reported QoL in two countries: the United Kingdom and the United States of America, with the aim to evaluate and compare measured QoL and the factors correlated. The QoL of people living in these two countries has been ranked among the top 20 in the world [10], yet they have different welfare and health care systems, which could impact QoL [11].

## Methods

### Study population

We used two large population-based studies: the Whitehall II study from the UK and the Western New York Health Study (*n* = 3,684) from the United States.

#### Whitehall II Study (WHS)

The UK participants in this report were sourced from the WHS, recruited from 20 civil service departments based in London, in 1985–1988 (phase 1). The initial response rate was 73%, and the final cohort consisted of 10,308 participants (3,413 women and 6,895 men). Follow-up screening was carried out in 1991–1993 (phase 3), 1997–1999 (phase 5), 2002–2004 (phase 7), and postal questionnaires were sent to participants in 1989 (phase 2), 1995 (phase 4), 2001 (phase 6) and 2006 (phase 8). The participation rates of the original cohort (*n* = 10,308) were 83, 76 and 68% at phases 3, 5, and 7, respectively. More details of this study can be found elsewhere [13, 14]. For the current analyses we used data from 6,472 white participants with available information who attended phase 5 of the WHS.

#### Western New York Health Study (WNYHS)

The US participants were taken from a sample of those enrolled as control participants in the WNYHS (detailed description can be found elsewhere [15]) In short, this is a series of case–control studies. Potential controls had to fulfil the following eligible criteria: (1) residents of Erie and Niagara counties: (2) age 35–79 years, (3) no cancer history. The participants were identified from two sources: (a) Department of Motor Vehicles of New York State for participants aged 35–64 years, (b) Health care financing administration (HCFA) lists for those aged 65–79 years. Six thousand eight hundred and thirty seven potential participants were identified, contacted and deemed eligible between 1996 and 2001. Of those 4,065 agreed to participate and were examined, for a participation rate of 59.5%. For the current analyses we used data from 3,684 white participants with available information, as previously described in detail [16].

### Short form-36 (SF-36)

The SF-36 is an instrument used in different populations to measure QoL and health status [17–19]. This form yields an 8-scale profile of functional health and well-being scores as well as psychometrically-based physical and mental health summary measures and a preference-based health utility index. Participants respond to 36 items aggregated to form subscales that measure the respondents’ impression of their health-related functioning in eight areas: physical functioning, role limitations due to physical problems, bodily pain, general health perceptions, vitality, social functioning, role limitations due to emotional problems and mental health. Scales are scored on a 0–100 scale, with higher scores indicating better QoL. The first four subscales indicate respondents’ physical health status (PCS), while the last four indicate mental health status (MCS). Factor analysis has been applied to the scales to create a physical and mental health component summary, which are standardized as *t* scores (mean of 50 and standard deviation of 10) and have higher reliability than the individual scales [20]. Therefore, in the current study, QoL assessment was based on these two component summaries (physical and mental).

### Correlates

All factors listed below were considered as potential correlates and were categorised separately by study to allow comparability between the two samples.

#### Socio-demographic factors

Age was categorised into ≤50, 51–60 and >60 years. Marital status was classified into married and unmarried. Socio-economic status (SES) was determined by individual’s income or their employment grade. In the WHS, participants’ last known civil service employment grade was used and divided in order of decreasing salary as follows: (1) administrative, (2) professional/executive and (3) clerical/support. In the Western New York Study, individuals’ annual household income was categorised into three groups of decreasing income: >$70,000, $30,000–70,000, and <$30,000. In order to make it comparable both groups were further categorized as either lowest, medium or highest SES.

#### Lifestyle factors

Body mass index (BMI) was calculated as weight (kg)/height (m^2^) and was classified into <25 (normal weight), 25–29.9 (overweight) and ≥30 (*obesity*)*.* Waist circumference was divided into tertiles based on the sample-specific distribution.

Smoking status was classified into current smoker and non-current smoker. Alcohol consumption was recorded in the previous week in the WHS, and in the last 30 days in the Western New York study; and divided into three categories: non-current drinker, low (below median) and high (above median) intake.

For physical activity, UK participants were asked to record the number of occasions/hours they had spent engaging in a series of specific activities over the previous 4 weeks. These activities were classified into light, moderate, or vigorous activities on the basis of their energy expenditure (metabolic equivalents). In the present study, the UK sample was categorized into two categories according to the energy expenditure: high vigorous activity (subjects who reported at least 1.5 h of vigorous activity per week); low vigorous activity (subjects who reported <1.5 h or absent of vigorous activity per week) [16]. In the US sample, physical activity was determined by participants completing the 7 day physical activity recall questionnaire used in the Stanford Five-City project [21]. For comparison, US participants were divided at the median into high and low physical activity group.

In the UK sample, sleep duration was elicited by the question “How many hours of sleep do you have on an average week night?” Response categories were 5 h or less, 6, 7, 8, and 9 h or more. In the US sample, sleep duration in the past week was ascertained with the 7-day physical activity recall questionnaire [21]. By the question “On the average, how many hours did you sleep each night during the last 5 weekday nights (Sunday–Thursday)?” To allow comparability response categories were collapsed into three groups: short sleep duration (<6 h), average (6–8 h), and long sleep duration (>8 h).

#### Comorbidity

In the UK sample, psychiatric morbidity including depressive symptoms was assessed with a modified general health questionnaire (GHQ) score. In the US sample, the presence of depressive symptoms was assessed by using the Center for Epidemiologic Studies Depression Scale (CES-D) [35]; participants were divided in two groups based on the cut point for major depressive symptoms (score ≥22).

In both samples, blood pressure was measured three times in the sitting position using a standard mercury sphygmomanometer by trained and certified technicians. The mean of the second and third measures were used in the analyses. Hypertension was defined as blood pressure ≥140/90 mmHg or regular use of antihypertensive medications. In both samples, fasting glucose concentrations were determined by glucose oxidase methods. Diabetes was defined either as fasting glucose ≥126 mg/dl or use of antidiabetic medications. Finally, in both samples personal medical history was obtained to determine the prevalence of cardiovascular disease (CVD), such as prior myocardial infarction, coronary artery bypass graft surgery, angioplasty or diagnosed angina pectoris, stroke, and use of cardiovascular medications.

### Statistical analysis

All analyses were conducted using the statistical package for social sciences (SPSS version 17.0). Descriptive analyses were performed for all selected variables. Covariates were selected based on previous publications. We computed age-adjusted and fully adjusted one way analyses of covariance (ANCOVA) by using selected variables as independent variables and the two SF-36 component summaries of (physical and mental health) as dependent variables, separately for the two studies and for women and men. All variables presented in the sections above were included in the multivariate model. The general linear model procedure was used to compare adjusted mean scores of physical and mental health across categories of selected variables and for *pairwise* comparisons rather than comparisons with a selected reference category. Adjustment for multiple comparisons was done by Bonferroni method.

## Results

### Characteristics of study participants

In the UK study, women constituted a smaller percent of the sample than in the US sample (28.3% vs. 51.7%) (Table 1). Compared to participants in the WNYHS, those in the WHS tended to be younger (mean age 55.8 vs. 58.7), less likely to be married (21.4% vs. 24.1%) and had fewer people in the lowest SES (11.4% vs. 33.7%). Levels of lifestyle factors were also different. The UK sample was leaner (mean BMI 26.1 vs. 28.2 kg/m^2^ and mean waist circumference 88.7 vs. 92.8 cm), smoked less (proportion of current smokers 10.7% vs. 14%) and had higher levels of physical activity.

**Table 1:** Baseline characteristics of the two populations included: WHS, London, UK (phase 5: 1997–1999); WNYHS, Buffalo, USA (1996–2001)

Variable	WHS (*n* = 6,472)	WNYHS (*n* = 3,684)
Mean age (years)	55.8 (6.1)	58.7 (11.9)
Women (%)	28.3	51.7
Not married (%)	21.4	24.1
Lowest SES^a^ (%)	11.4	33.7
Sleep duration (%) [h]		
<6	7.5	13.7
6–8	91.1	80.0
>8	1.4	6.6
Body mass index (kg/m^2^)	26.1 (3.9)	28.2 (5.5)
Waist circumference (cm)	88.7 (11.8)	92.8 (14.9)
Current smoker (%)	10.7	14.0
Current drinker (%)	86.0	67.2
Daily alcohol consumption^b^ (U)	2.4 (2.2)	0.63 (1.5)
Low physical activity (%)	55.9	50.2
SF-36 score		
Physical	51.2 (8.0)	48.6 (9.3)
Mental	51.1 (9.4)	53.1 (8.3)
Depressive symptoms (%)	12.4	9.8
Systolic blood pressure (mmHg)	122.9 (16.3)	122.2 (16.8)
Diastolic blood pressure (mmHg)	77.5 (10.6)	72.7 (9.9)
Hypertension^c^ (%)	29.2	35.6
Diabetes^d^ (%)	2.5	8.5
CVD (%)	15.5	13.6

Data are expressed as the mean (SD) or as percentages

^a^SES (socio-economic status) based on the lowest employment grade in the WHS and lowest annual household income in the WNYHS

^b^Computed among current drinkers only

^c^Defined as blood pressure ≥140/90 mmHg or regular use of antihypertensive medications

^d^Defined as fasting glucose ≥126 mg/dl (≥7.0 mmol/l) or use of antidiabetic medications

The WNYHS participants tended to drink less (fewer units of alcohol and smaller proportion of drinkers) and had a greater proportion of “short” and “long” sleepers and a higher proportion of participants suffering from hypertension and diabetes. The US sample had a lower prevalence of depressive symptoms and a lower proportion of participants with established CVD (13.6% vs. 15.5%).

### Correlates of quality of life (QoL)

#### SF-36 scores

Measured physical QoL tended to be higher in the UK sample (51.2 vs. 48.6) while mental QoL was higher in the US sample (53.1 vs. 51.1).

#### Age-adjusted mean scores

When we evaluated the associations between age-adjusted mean SF-36 physical and mental health scores, several factors were significantly and consistently related to the SF-36 scores (QoL) (Tables 2, 3).

**Table 2:** Age-adjusted mean scores (SE) of the SF-36 components summaries by gender and selected correlates: WHS

Variable	*N*	Mean (SE)**	*P*	Mean (SE)**	*P*	*N*	Mean (SE)**	*P*	Mean (SE)**	*P*
		Men (*n* = 4,643)					Women (*n* = 1,829)			
		Physical		Mental			Physical		Mental	
Age (years)										
≤50	973	53.1 (0.22)	<0.001	48.7 (0.28)	0.001	346	51.5 (0.51)	<0.001	46.5 (0.55)	<0.001
51–60	2,301	52.2 (0.15)		51.3 (0.18)		864	49.2 (0.33)		49.2 (0.35)	
>60	1,263	50.7 (0.20)		54.4 (0.25)		619	47.4 (0.39)		52.5 (0.42)	
Sleep (h)										
<6	285	49.4 (0.41)	<0.001	46.6 (0.51)	0.001	177	45.9 (0.70)	0.001	43.7 (0.74)	0.001
6–8	4,200	52.2 (0.11)		52.0 (0.13)		1,533	49.5 (0.24)		50.4 (0.25)	
>8	52	50.5 (0.97)		50.8 (1.20)		37	45.3 (1.54)		49.1 (1.63)	
Marital status										
Married	3,831	52.0 (0.11)	0.91	52.1 (0.14)	0.001	1,074	48.8 (0.29)	0.22	50.6 (0.31)	<0.001
Not married	675	52.0 (0.27)		49.3 (0.34)		660	49.4 (0.38)		48.8 (0.40)	
Socioeconomic status										
Lowest	202	51.5 (0.52)	0.011	48.2 (0.64)	<0.001	534	48.8 (0.44)	0.005	49.6 (0.47)	0.045
Medium	2,335	52.3 (0.14)		51.4 (0.20)		403	48.6 (0.32)		49.4 (0.35)	
Highest	1,885	51.6 (0.16)		52.0 (0.17)		875	50.4 (0.48)		50.9 (0.52)	
BMI (kg/m^2^)										
<25	1,490	52.7 (0.18)	<0.001	51.6 (0.23)	0.54	657	50.9 (0.36)	<0.001	48.9 (0.41)	0.17
25–29.9	1,698	52.2 (0.16)		51.7 (0.21)		485	49.4 (0.42)		49.9 (0.48)	
≥30	431	49.6 (0.33)		51.2 (0.42)		260	46.0 (0.57)		50.1 (0.65)	
Waist (tertile)										
1 (lowest)	638	53.1 (0.27)	<0.001	51.9 (0.34)	0.41	872	50.5 (0.31)	<0.001	49.1 (0.36)	0.39
2	1,264	52.6 (0.19)		51.6 (0.25)		250	48.3 (0.58)		49.3 (0.66)	
3 (highest)	1,313	50.9 (0.19)		51.3 (0.24)		184	46.1 (0.68)		50.3 (0.78)	
Smoking status										
Non-current smoker	4,205	52.1 (0.11)	<0.001	51.6 (0.14)	0.74	1,558	49.2 (0.24)	0.08	50.1 (0.26)	0.001
Current smoker	426	50.4 (0.34)		51.5 (0.43)		262	48.1 (0.60)		47.8 (0.64)	
Drinking status										
Non-current drinker	465	51.1 (0.33)	0.022	50.5 (0.41)	0.016	432	46.5 (0.47)	<0.001	49.5 (0.51)	0.14
Low	1,900	52.1 (0.16)		51.7 (0.41)		983	49.4 (0.30)		50.2 (0.33)	
High	2,245	52.0 (0.15)		51.8 (0.19)		384	51.1 (0.48)		49.1 (0.52)	
Physical activity										
High	2,255	52.6 (0.15)	<0.001	52.1 (0.19)	0.001	598	50.6 (0.39)	<0.001	50.6 (0.42)	0.014
Low	2,388	51.4 (0.14)		51.2 (0.18)		1,231	48.3 (0.27)		49.4 (0.29)	
Depressive symptoms										
No	4,041	52.1 (0.11)	0.016	53.1 (0.12)	<0.001	1,548	49.4 (0.24)	0.002	51.8 (0.23)	<0.001
Yes	539	51.3 (0.31)		40.0 (0.34)		251	47.4 (0.61)		37.3 (0.56)	
Hypertension										
No	3,000	52.3 (0.13)	<0.001	51.8 (0.16)	0.013	1,159	49.8 (0.28)	<0.001	49.2 (0.31)	0.001
Yes	1,265	51.1 (0.20)		51.1 (0.25)		453	47.9 (0.46)		51.2 (0.50)	
Diabetes										
No	4,058	52.1 (0.11)	<0.001	51.7 (0.14)	0.17	1,519	49.6 (0.24)	0.48	49.7 (0.27)	0.92
Yes	117	48.6 (0.65)		50.5 (0.82)		28	48.3 (1.78)		49.9 (1.99)	
Cardiovascular dis.										
No	3,931	52.4 (0.11)	<0.001	51.8 (0.14)	<0.001	1,531	49.6 (0.25)	<0.001	49.7 (0.27)	0.33
Yes	704	49.6 (0.27)		50.4 (0.34)		295	46.0 (0.57)		50.3 (0.61)	

Estimated marginal means adjusted for age

** Higher scores indicate better health and *functioning* (*except for sleep*). *P* value indicates the significant linear trend (*P* ≤ 0.05)

**Table 3:** Age-adjusted mean scores (SE) of the SF-36 components summaries by gender and selected correlates: WNYHS

Variable	*N*	Mean (SE)**	*P*	Mean (SE)**	*P*	*N*	Mean (SE)**	*P*	Mean (SE)**	*P*
		Men (*n* = 1,781)					Women (*n* = 1,903)			
		Physical		Mental			Physical		Mental	
Age (years)										
≤50	463	52.0 (0.39)	<0.001	52.2 (0.36)	<0.001	632	50.4 (0.38)	<0.001	50.8 (0.34)	<0.001
51–60	321	49.8 (0.47)		53.9 (0.36)		435	48.9 (0.46)		52.1 (0.41)	
>60	924	47.4 (0.28)		54.6 (0.25)		739	45.8 (0.35)		53.9 (0.31)	
Sleep (h)										
<6	245	47.5 (0.53)	<0.001	52.4 (0.49)	0.003	231	44.9 (0.62)	<0.001	50.6 (0.56)	0.002
6–8	1,337	49.6 (0.23)		54.2 (0.21)		1,459	48.9 (0.25)		52.7 (0.22)	
>8	120	46.6 (0.76)		53.2 (0.71)		112	45.8 (0.89)		52.1 (0.80)	
Marital status										
Married	1,426	49.3 (0.22)	0.04	54.3 (0.20)	<0.001	1,253	48.4 (0.27)	0.19	52.9 (0.24)	<0.001
Not married	276	48.2 (0.50)		51.3 (0.46)		549	47.7 (0.41)		51.1 (0.37)	
Socioeconomic status										
Lowest	344	47.6 (0.39)	<0.001	52.6 (0.36)	<0.001	325	46.9 (0.42)	<0.001	51.3 (0.38)	0.005
Medium	797	49.2 (0.29)		53.9 (0.27)		736	48.7 (0.35)		53.9 (0.27)	
Highest	492	51.3 (0.46)		55.1 (0.43)		596	50.5 (0.54)		55.1 (0.46)	
BMI (kg/m^2^)										
<25	396	50.0 (0.41)	<0.001	53.3 (0.39)	0.16	646	50.6 (0.36)	<0.001	51.9 (0.33)	0.10
25–29.9	773	49.7 (0.30)		54.2 (0.28)		568	49.3 (0.38)		52.9 (0.36)	
≥30	496	47.6 (0.37)		53.5 (0.35)		531	44.5 (0.39)		52.4 (0.37)	
Waist (tertile)										
1 (Lowest)	234	50.9 (0.54)	<0.001	53.8 (0.51)	0.64	964	50.6 (0.28)	<0.001	52.4 (0.27)	0.97
2	680	50.3 (0.31)		54.0 (0.30)		461	47.9 (0.41)		52.3 (0.39)	
3 (Highest)	751	47.8 (0.30)		53.6 (0.28)		321	43.2 (0.49)		52.3 (0.47)	
Smoking status										
Non-current smoker	1,481	49.6 (0.22)	<0.001	54.0 (0.20)	0.023	1,543	48.3 (0.24)	0.09	52.5 (0.22)	0.05
Current smoker	224	46.0 (0.56)		52.7 (0.53)		259	47.2 (0.59)		51.4 (0.53)	
Drinking status										
Non-current drinker	456	47.4 (0.40)	<0.001	52.5 (0.37)	<0.001	740	46.2 (0.36)	<0.001	51.7 (0.32)	0.027
Low	215	49.1 (0.58)		53.1 (0.54)		330	48.5 (0.53)		52.4 (0.45)	
High	1,080	49.8 (0.26)		54.5 (0.24)		822	49.8 (0.34)		52.9 (0.30)	
Physical activity										
High	937	50.7 (0.27)	<0.001	54.3 (0.25)	0.002	826	50.0 (0.33)	<0.001	52.5 (0.30)	0.47
Low	768	47.1 (0.30)		53.2 (0.28)		977	46.6 (0.30)		52.2 (0.27)	
Depressive symptoms										
No	1,445	49.7 (0.21)	<0.001	54.9 (0.18)	<0.001	1,446	48.9 (0.25)	<0.001	54.0 (0.19)	<0.001
Yes	115	44.2 (0.76)		42.8 (0.64)		198	44.0 (0.66)		41.7 (0.52)	
Hypertension										
No	1,037	49.5 (0.26)	0.010	53.9 (0.24)	0.46	1,239	49.2 (0.27)	<0.001	52.4 (0.25)	0.71
Yes	671	48.4 (0.33)		53.6 (0.31)		567	45.8 (0.42)		52.2 (0.38)	
Diabetes										
No	1,347	49.5 (0.23)	<0.001	53.9 (0.21)	0.37	1,530	48.8 (0.24)	<0.001	52.4 (0.22)	0.25
Yes	285	47.1 (0.50)		53.4 (0.47)		167	43.6 (0.73)		51.6 (0.67)	
CVD										
No	1,343	49.8 (0.23)	<0.001	54.0 (0.21)	0.022	1,693	48.4 (0.23)	<0.001	52.4 (0.21)	0.14
Yes	365	46.5 (0.45)		52.1 (0.42)		112	44.5 (0.92)		51.2 (0.83)	

Estimated marginal means adjusted for age

** Higher scores indicate better health and functioning (*except for sleep*). *P* value indicates the significant linear trend (*P* ≤ 0.05)

#### Fully-adjusted mean scores

In analyses where we further adjusted for the variables included, age, sleep duration and presence of depressive symptoms appeared as the most consistent and relevant correlates of QoL in both populations and in men and women (Tables 4, 5). Specifically, increasing age was associated with poorer physical health but with higher mental health scores (*P* < 0.001) in both samples.

**Table 4:** Fully-adjusted mean scores (SE) of the SF-36 components summaries by gender and selected correlates: WHS

Variable	*N*	Mean (SE)**	*P*	Mean (SE)**	*P*	*N*	Mean (SE)**	*P*	Mean (SE)**	*P*
		Men (*n* = 4,643)^a^					Women (*n* = 1,829)^a^			
		Physical		Mental			Physical		Mental	
Age (years)										
≤50	973	50.2 (0.67)	<0.001	42.7 (0.77)	<0.001	346	47.8 (1.38)	0.003	41.2 (1.35)	<0.001
51–60	2,301	49.6 (0.63)		44.9 (0.75)		864	46.5 (1.33)		42.5 (1.30)	
>60	1,263	48.3 (0.66)		47.5 (0.78)		619	45.2 (1.35)		45.2 (1.31)	
Sleep (h)										
<6	285	48.6 (0.66)	0.019	43.5 (0.77)	<0.001	177	46.4 (1.33)	0.001	40.1 (1.30)	<0.001
6–8	4,200	49.9 (0.51)		46.4 (0.60)		1,533	49.0 (1.14)		45.3 (1.12)	
>8	52	49.7 (1.22)		45.2 (1.45)		37	44.1 (2.16)		43.5 (2.11)	
Marital status										
Married	3,831	49.1 (0.63)	0.10	45.4 (0.75)	0.11	1,074	46.0 (1.31)	0.038	43.5 (1.28)	0.043
Not married	675	49.7 (0.67)		44.7 (0.79)		660	47.1 (1.33)		42.4 (1.30)	
Socioeconomic status										
Lowest	202	48.6 (0.79)	0.88	44.3 (0.91)	0.23	534	46.3 (1.32)	0.15	43.3 (1.30)	0.28
Medium	2,335	48.5 (0.63)		45.5 (0.73)		403	45.6 (1.38)		43.8 (1.36)	
Highest	1,885	48.4 (0.61)		45.4 (0.91)		875	45.1 (1.27)		42.9 (1.25)	
BMI (kg/m^2^)										
<25	1,490	50.2 (0.65)	<0.001	44.8 (0.76)	0.45	657	48.4 (1.34)	<0.001	42.7 (1.31)	0.75
25–29.9	1,698	50.0 (0.64)		44.9 (0.76)		485	47.1 (1.35)		42.9 (1.32)	
≥30	431	47.9 (0.69)		45.4 (0.82)		260	44.0 (1.37)		43.2 (1.34)	
Smoking status										
Non-current smoker	4,205	50.3 (0.61)	<0.001	44.8 (0.72)	0.25	1,558	46.8 (1.24)	0.52	43.7 (1.21)	0.07
Current smoker	426	48.4 (0.72)		45.4 (0.85)		262	46.3 (1.45)		42.2 (1.42)	
Drinking status										
Non-current drinker	465	48.1 (0.69)	0.22	45.0 (0.80)	0.84	432	43.5 (1.34)	<0.001	43.7 (1.32)	0.59
Low	1,900	48.7 (0.62)		45.0 (0.72)		983	46.0 (1.39)		43.0 (1.27)	
High	2,245	48.8 (0.62)		45.1 (0.72)		384	47.5 (1.35)		43.3 (1.33)	
Physical activity										
High	2,255	49.9 (0.65)	<0.001	45.2 (0.77)	0.20	598	47.3 (0.36)	0.005	43.1 (1.33)	0.56
Low	2,388	48.9 (0.63)		44.9 (0.75)		1,231	45.7 (0.28)		42.8 (1.25)	
Depressive symptoms										
No	4,041	49.5 (0.62)	0.55	51.4 (0.74)	<0.001	1,548	47.1 (1.27)	0.14	50.0 (1.25)	<0.001
Yes	539	49.3 (0.69)		38.7 (0.82)		251	45.9 (1.42)		35.9 (1.39)	
Hypertension										
No	3,000	49.4 (0.64)	0.80	45.2 (0.78)	0.46	1,159	46.7 (1.31)	0.58	41.8 (1.28)	0.001
Yes	1,265	49.4 (0.66)		44.9 (0.74)		453	46.3 (1.37)		44.1 (1.34)	
Diabetes										
No	4,058	50.4 (0.52)	0.012	45.1 (0.61)	0.87	1,519	46.4 (0.83)	0.91	42.8 (0.81)	0.87
Yes	117	48.4 (0.92)		45.0 (1.09)		28	46.6 (2.18)		43.1 (2.13)	
CVD										
No	3,931	50.3 (0.65)	<0.001	45.0 (0.73)	0.85	1,531	46.9 (1.35)	0.011	43.4 (1.32)	0.44
Yes	704	48.5 (0.64)		45.1 (0.75)		295	46.1 (1.32)		42.8 (1.29)	

Estimated marginal means adjusted for age

** Higher scores indicate better health and functioning (except for sleep). *P* value indicates the significant linear trend (*P* ≤ 0.05)

^a^Male *R* ^2^ = 0.32, female *R* ^2^ = 0.27

**Table 5:** Fully-adjusted mean scores (SE) of the SF-36 components summaries by gender and selected correlates: WNYHS

Variable	*N*	Mean (SE)**	*P*	Mean (SE)**	*P*	*N*	Mean (SE)**	*P*	Mean (SE)**	*P*
		Men (*n* = 1,781)***					Women (*n* = 1,903)***			
		Physical		Mental			Physical		Mental	
Age (years)										
≤50	463	46.0 (0.71)	0.002	45.6 (0.62)	<0.001	632	45.0 (0.87)	0.029	45.3 (0.77)	<0.001
51–60	321	44.6 (0.76)		47.4 (0.66)		435	44.2 (0.87)		47.0 (0.77)	
>60	924	44.1 (0.64)		48.9 (0.57)		739	43.3 (0.82)		48.6 (0.73)	
Sleep (h)										
<6	245	44.8 (0.74)	0.032	47.2 (0.65)	0.46	231	43.1 (0.92)	<0.001	46.2 (0.81)	0.23
6–8	1,337	45.9 (0.59)		47.7 (0.52)		1,459	45.7 (0.75)		46.9 (0.66)	
>8	120	44.1 (0.97)		47.0 (0.86)		112	43.7 (1.15)		47.9 (1.01)	
Marital status										
Married	1,426	44.7 (0.60)	0.46	47.9 (0.53)	0.04	1,253	44.0 (0.79)	0.71	47.6 (0.70)	0.012
Not married	276	45.1 (0.77)		46.8 (0.68)		549	44.2 (0.86)		46.4 (0.76)	
Socioeconomic status										
Lowest	344	43.6 (0.67)	<0.001	46.7 (0.59)	0.07	325	42.7 (0.82)	0.003	46.9 (0.72)	0.90
Medium	797	44.6 (0.65)		47.2 (0.58)		736	44.4 (0.82)		47.1 (0.72)	
Highest	492	46.7 (0.78)		48.0 (0.69)		596	45.3 (0.95)		46.9 (0.84)	
BMI (kg/m^2^)										
<25	396	45.3 (0.73)	0.033	47.0 (0.65)	0.42	646	46.1 (0.86)	<0.001	46.3 (0.76)	0.06
25–29.9	773	45.3 (0.67)		47.6 (0.60)		568	45.0 (0.86)		47.4 (0.75)	
≥30 (C)	496	43.9 (0.67)		47.3 (0.59)		531	41.3 (0.82)		47.3 (0.73)	
Smoking status										
Non-current smoker	1,481	46.3 (0.60)	<0.001	47.5 (0.53)	0.57	1,543	44.6 (0.75)	0.13	47.2 (0.66)	0.43
Current smoker	224	43.5 (0.78)		47.1 (0.69)		259	43.6 (0.92)		46.8 (0.81)	
Drinking status										
Non-current drinker	456	44.1 (0.68)	0.031	46.9 (0.60)	0.033	740	43.2 (0.82)	0.013	47.3 (0.72)	0.49
Low	215	45.3 (0.84)		47.0 (0.55)		330	44.5 (0.90)		46.6 (0.79)	
High	1,080	45.4 (0.63)		48.0 (0.56)		822	44.7 (0.83)		47.1 (0.73)	
Physical activity										
High	937	46.3 (0.66)	<0.001	47.8 (0.59)	0.006	826	45.7 (0.82)	<0.001	47.2 (0.73)	0.20
Low	768	43.5 (0.64)		46.8 (0.57)		977	42.6 (0.79)		46.7 (0.70)	
Depressive symptoms										
No	1,445	46.9 (0.57)	<0.001	53.2 (0.51)	<0.001	1,446	45.7 (0.74)	<0.001	53.2 (0.67)	<0.001
Yes	115	42.9 (0.87)		41.4 (0.77)		198	42.6 (0.95)		40.9 (0.84)	
Hypertension										
No	1,037	45.5 (0.65)	0.006	47.3 (0.57)	0.77	1,239	45.0 (0.81)	0.001	46.7 (0.71)	0.30
Yes	671	44.3 (0.67)		47.5 (0.59)		567	43.3 (0.84)		47.2 (0.74)	
Diabetes										
No	1,347	45.5 (0.61)	0.006	47.3 (0.54)	0.83	1,530	44.6 (0.72)	0.29	47.1 (0.63)	0.85
Yes	285	44.4 (0.76)		47.3 (0.67)		167	43.7 (1.03)		46.9 (0.91)	
CVD										
No	1,343	43.7 (0.74)	<0.001	47.2 (0.65)	0.63	1,693	42.6 (1.16)	0.003	46.4 (1.02)	0.19
Yes	365	46.1 (0.61)		47.4 (0.54)		112	45.7 (0.65)		47.6 (0.58)	

Estimated fully adjusted marginal means

** Higher scores indicate better health and functioning (*except for sleep*). *P* value indicates the significant linear trend (*P* ≤ 0.05)

^a^Male *R* ^2^ = 0.25, female *R* ^2^ = 0.22

Sleep duration had an inverted *u* shaped significant association with the SF-36 scores. In fact, both short and long duration of sleep were consistently associated with lower scores in both the UK and US sample. This association was significant for both mental and physical SF-36 scores in men and women in the UK sample while in the US sample sleep duration tended to only affect physical QoL.

A contrasting scenario was observed for the presence of depressive symptoms, which was significantly associated with both physical and mental QoL in both men and women of the US sample but only with mental QoL scores in the UK participants.

Other factors were significantly associated with either one dimension of QoL in both populations or with both but within a single population or only in men or women and overall, lifestyle variables and co-morbidities were more associated with the physical than the mental QoL component (Tables 4, 5).

## Discussion

Overall we found that levels of physical QoL tended to be higher in the UK population while mental QoL was higher in the US group perhaps reflecting intrinsic differences present in the two populations selected. Beyond the levels of QoL, we found consistent findings from this cross-cultural comparison of correlates of QoL, with age, sleep duration and presence of depressive symptoms being the most consistent and relevant correlates.

### Socio-demographic correlates

Of the correlates evaluated, the most consistent finding was that increasing age was strongly associated with poorer physical QoL but with significantly higher mental QoL in both men and women from both the UK and US samples. The reduced physical score in the older age group can be explained by a general deterioration in body functions and capabilities; however the improved mental health score might be due to better coping abilities and adaptation in this age group [22]. In fact, this finding supports previous studies suggesting that older people tend to have internal mechanisms available to accommodate better to hardship or negative circumstances than those who are younger [23].

With regard to SES, people from a lower socio-economic group had lower scores of QoL in general. However, this trend was only significant in the US sample in the fully-adjusted models, which could mean that the gradient seen in the UK sample in the only age-adjusted models is ‘explained’ by the other correlates in the analyses. Furthermore, this could also be attributed to the differential classification of social status in our study because we divided the UK sample based on their employment grades, while we used household income as a measure of SES for the US sample. In addition, the different nature of the two populations (occupational vs. communitywide sample) is likely to play a role. It may also be that SES is less strongly associated with QoL in the UK because the magnitude of differences in access to health care by SES might be lower than in the US.

### Lifestyle factors

In the present study, lifestyle variables were more strongly associated with the physical rather than the mental component of QoL. This is somewhat inconsistent with previous research, which suggests, for example, that regular physical exercise may improve mental health wellbeing as well as physical health [24–28]. One possible explanation may be that our classification of physical activity levels into high and low, might not fully capture the true effect of physical activity. The cross-sectional nature of the present analyses does not allow detection of the causal direction of the association for example whether physical activity might have a longer-term protective effect on mental QoL.

Our results show that people who sleep between 6 and 8 h/day tend to have both better physical and mental health scores than those who slept on average <6 or more than 8 h/day. This finding is supported by a growing body of evidence where short (<6 h) and long duration of sleep (>8 h) are related to poorer self perceived mental and physical health, as well as increased risk of adverse health outcomes and higher total mortality [16, 29–31]. This finding highlights the need to pay closer attention to the societal changes in sleep patterns that have occurred in the last years and which might have a substantial role in the current global epidemics of cardiometabolic disorders.

With regard to drinking habits, in the current study, non-current drinkers of both genders and countries reported consistently poorer physical and mental QoL scores than current drinkers. Non-current drinkers may include subjects who no longer drink because of pre-existing diseases, which confounds the relationship between health status and alcohol consumption [15, 32, 33].

Current smoking appears to be strongly related to physical functioning in men in both studies, but there appears to be no strong evidence of an association with physical health in women or with mental health in either sex. The most likely explanation of the gender difference in the association with physical health in these two, middle-aged cohorts will be the strength of the exposure. Men are more likely to have been heavy smokers and have smoked for longer than women.

### Co-morbidities

Depressive symptoms were strongly associated with poorer mental QoL in both samples and genders and with poorer physical QoL in American men and women. Presence of CVD was consistently associated with poorer physical health in both samples and genders, while prevalent hypertension seemed to affect only the physical QoL of US participants. Diabetes on the other hand only affected the physical QoL in men of both samples, perhaps reflecting the gender distribution in prevalence of diabetes in the two populations -and the level of severity. As with the lack of effect of physical activity on mental QoL, it is possible that the cross-sectional nature of the present analyses does not allow us to detect longer-term deleterious effects that co-morbidities might have on mental QoL.

## Limitations and strengths

Despite a large amount of research on the measurement and validity of health related QoL, there have only been a handful of studies on factors associated with QoL [12, 34]. The present study attempted to address this issue by examining two well-characterised populations. By performing a cross-cultural comparison, we attempted to further establish the correlation between certain selected variables and QoL. To our knowledge, our study is the first of its kind to investigate determinants of QoL using the standardised SF-36 questionnaire while taking into account other covariates in a cross-cultural setting. Beyond this, different limitations in the present study warrant consideration. Firstly, while the cross-cultural design of this study allowed us to examine the associations between QoL and multiple factors, it does not allow us to establish the causality and temporality of the observed relationships. Secondly, both samples were also limited to Caucasians, and originated from developed western societies; which might reduce the generalizability of our findings to different ethnic backgrounds and socio-economic settings. Thirdly, although we have included a comprehensive range of factors associated with QoL, additional key factors (e.g. stress, social support, job satisfaction, social integration, personality) have not been measured in both of these samples, and we were not able to compare them between our included populations. Fourthly, given the cross-sectional nature of the study, it is not possible to disentangle the chronological order or causal nature of the associations found, nor to fully understand the effects of cumulative experience of factors evaluated across the lifecourse. Lastly, questions asked in both studies varied slightly which might lead to discrepancies, challenging the comparability of the two populations.

## Conclusions

In conclusion, consistent findings from this cross-cultural comparison between two populations from the UK and US corroborate the multifaceted nature of measures of QoL. Increasing age was associated with poorer physical health but with higher mental health scores. Lifestyle and co-morbidity factors mainly affected the physical health component and had little impact on the mental health component. These are novel findings that warrant further consideration and suggest additional aspects to consider when trying to improve or maintain the QoL of a population. Beyond our results, larger evaluations and comparisons in different populations are warranted to better understand crucial factors impacting QoL, factors that could be targeted to improve health outcomes in populations.
